# Use of microcarriers in Mobius^® ^CellReady bioreactors to support growth of adherent cells

**DOI:** 10.1186/1753-6561-7-S6-P95

**Published:** 2013-12-04

**Authors:** Michael McGlothlen, Donghui Jing, Christopher Martin, Michael Phillips, Robert Shaw

**Affiliations:** 1 EMD Millipore Corporation, 80 Ashby Rd, Bedford MA 01730, USA

## Abstract

The Mobius^® ^CellReady bioreactor product platform incorporates novel disposable technologies that provide optimal performance for suspension mammalian cell culture. Here we show the utility of EMD Millipore's 3L and 50L CellReady single use bioreactors for the cultivation of adherent mammalian cells on microcarriers. Cytodex^3^^® ^and Solohill^® ^collagen microcarriers were first tested in a mixing study to assess feasibility. We evaluated the normalized mixing speed required in the 3L and 50L to achieve a suspension of the microcarriers and enable growth of the cells.

## Mixing

Manufacturer specifications show Cytodex^3^^® ^and Solohill^® ^microcarriers to be similar in density and size. Working with this assumption, mixing studies where performed using the Cytodex^3^^® ^microcarriers in 3L Mobius^® ^CellReady and Solohill^® ^Collagen coated in 50L single use bioreactor to determine the slowest agitation speed or the just suspended mixing power inputs (P/V)_js_, required to fully suspend the microcarriers so that the beads are equally distributed in the bioreactor.

Microcarrier distribution was assessed by sampling the bioreactor at varying depths. Then the dry weight of the microcarrier was used to determine the% relative sample weight to the target weight.

## Mixing Results

Data show the (P/V)_js _to be ~0.6W/m^3 ^in both the 3L and 50L single use bioreactors 
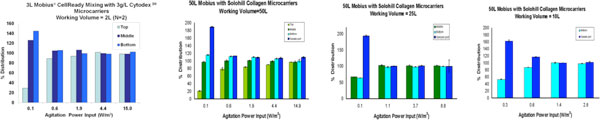


100% distribution corresponds to the theoretical concentration of microcarriers, which is 3g/L Cytodex^3^^® ^in 3L bioreactor and 15g/L Solohill^® ^Collagen microcarriers in 50L bioreactor

## Cell Growth

Initial cell culture runs were performed with MDCK and Human Mesenchymal Stem Cells (hMSCs) to evaluate the bioreactor agitation to support cell growth in the 3L Mobius^® ^CellReady single use bioreactor. The conditions that showed the best performance could then scaled to the 50L Mobius^® ^bioreactor.

1. Cultured MDCK cells on Cytodex^3^^® ^microcarriers grew to a peak cell density of ~1e6cells/mL using a power input of 0.6W/m^3 ^with a 2L working volume after 3 days.

2. Cultured hMSCs on Solohill^® ^microcarriers grew to a maximum total cell number of 6e6 cells using power input of 0.6-0.8W/m^3 ^with a 2.4L working volume after 12 days.

## Conclusions

1. Data from the mixing experiments demonstrate the just suspended mixing power input was determined to be ~0.6W/m^3^.

2. Cell growth experiments with hMSCs demonstrate comparable cell growth in the 3L and 50L Mobius^® ^CellReady bioreactor with total number of hMSCs reaching 4e8 and 9e9 cells after 12 days at a agitation power input of 0.6-0.8W/m^3^

3. Initial cell growth experiments with adherent MDCK cells demonstrate an agitation power/volume input of 0.6W/m^3 ^may provide the best performance for cell growth with peak cell densities ~1.0e6 cells/mL after 3 days

4. Comparable MDCK cell growth is observed:

Mobius^® ^CellReady Bioreactor 3L

Mobius^® ^CellReady Bioreactor 50L

Rocking Bioreactor 20L

**Figure 1:**
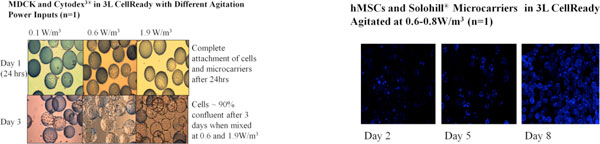
Illustrates the attachment of MDCK and hMSCs to Cytodex^3^^® ^and Solohill^® ^microcarriers

**Figure 2:**
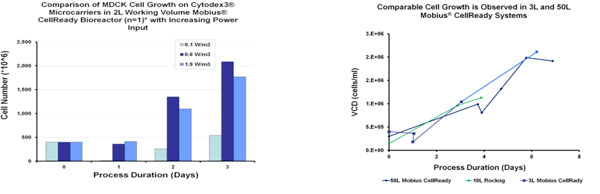
compares the viable cell density of MDCK cells at increasing power/volume impeller inputs and different bioreactors

**Figure 3:**
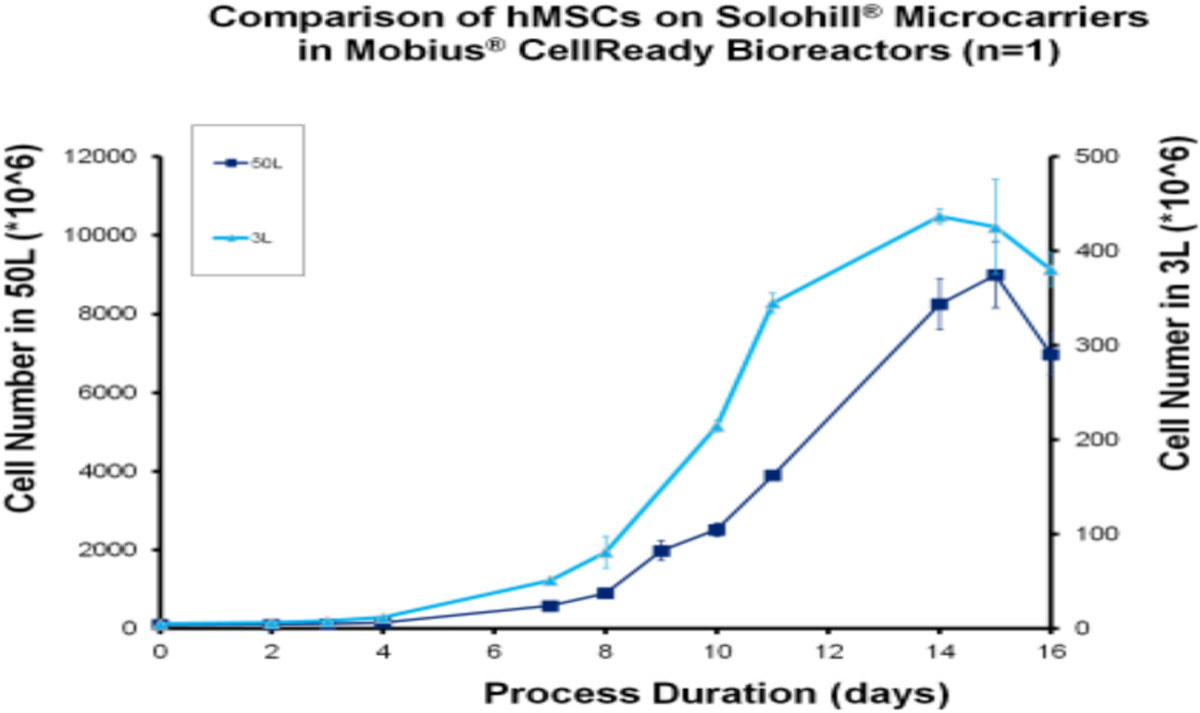
shows the viable cell density of hMSCs in the 3L and 50L Mobius^® ^CellReady Bioreactor

**Figure 4:**
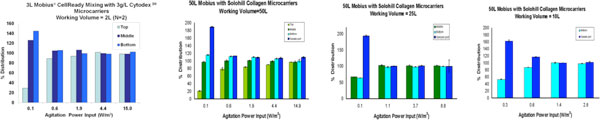


**Table 1:** 

Physical Characteristics of Microcarriers		
Microcarrier	Cytodex ^3 ®^	Solohill^® ^Collagen Coated

Density (g/ml)	1.04	1.03

Hydrated Size (µm)	141-211	125-212

Concentration (g/ml)	3	15


**Table 2:** 

MDCK/ Cytodex ^3 ® ^Microcarriers Process Table		
**Variable**	**Value**	

Cells	MDCK	hMSCs

Inoculation Density	4e5 cells/mL	5e3 cells/mL

Substrate	Cytodex ^3 ®^	Solohill^®^

Growth Media	DMEM w/ 4.5g/L Glucose, 2% FBS, 1% NEAA and 2mM L-Glutamine	DMEM low glucose, 10% FBS, 8ng/ml bFGF, 2mM Glutamine, 1X Pen/Strep

pH	7	NA

DO (% Saturation)	45	NA

Feed 1	Day 1: 100% Growth Media	Day 6: 1000ml low glucose fresh medium

Feed 2	Day 3: Drain 50% of the working volume and reefed with equal volume of Growth Media	Day 9: 400ml high glucose fresh medium

Batch Duration	7 days	12 days


